# Factors influencing smoking behaviour of online ride-hailing drivers in China: a cross-sectional analysis

**DOI:** 10.1186/s12889-021-11366-8

**Published:** 2021-07-06

**Authors:** Xinlin Chen, Xuefei Gu, Tingting Li, Qiaoyan Liu, Lirong Xu, Bo Peng, Nina Wu

**Affiliations:** 1 School of Public Health, Capital Medical University, No 10 Xitoutiao, Youanmenwai Street, Fengtai District, Beijing, China; 2 China National Health Development Research Center, Beijing, China; 3 China Rehabilitation Research Center, Beijing, China; 4 Institute of Medical Information, China Academy of Chinese Medical Sciences, Beijing, China

**Keywords:** Smoking, Online ride-hailing, Drivers, Influencing factors, China

## Abstract

**Background:**

Online ride-hailing is a fast-developing new travel mode. However, tobacco control policies on its drivers remain underdeveloped. This study aims to reveal the status and determine the influencing factors of ride-hailing drivers’ smoking behaviour to provide a basis for the formulation of tobacco control policies.

**Methods:**

We derived our cross-sectional data from an online survey of full-time ride-hailing drivers in China. We used a survey questionnaire to collect variables, including sociodemographic and work-related characteristics, health status, health behaviour, health literacy and smoking status. Finally, we analysed the influencing factors of current smoking by conducting chi-square test and multivariate logistic regression.

**Results:**

A total of 8990 ride-hailing drivers have participated in the survey, in which 5024 were current smokers, accounting to 55.9%. Nearly one-third of smokers smoked in their cars (32.2%). The logistic regression analysis results were as follows: male drivers (OR = 0.519, 95% CI [0.416, 0.647]), central regions (OR = 1.172, 95% CI [1.049, 1.309]) and eastern regions (OR = 1.330, 95% CI [1.194, 1.480]), working at both daytime and night (OR = 1.287, 95% CI [1.164, 1.424]) and non-fixed time (OR = 0.847, 95% CI [0.718, 0.999]), ages of 35–54 years (OR = 0.585, 95% CI [0.408, 0.829]), current drinker (OR = 1.663, 95% CI [1.526, 1.813]), irregular eating habits (OR = 1.370, 95% CI [1.233, 1.523]), the number of days in a week of engaging in at least 10 min of moderate or vigorous exercise ≥3 (OR = 0.752, 95% CI [0.646, 0.875]), taking the initiative to acquire health knowledge occasionally (OR = 0.882, 95% CI [0.783, 0.992]) or frequently (OR = 0.675, 95% CI [0.591, 0.770]) and underweight (OR = 1.249, 95% CI [1.001, 1.559]) and overweight (OR = 0.846, 95% CI [0.775, 0.924]) have association with the prevalence of current smoking amongst online ride-hailing drivers.

**Conclusion:**

The smoking rate of ride-hailing drivers was high. Sociodemographic and work-related characteristics and health-related factors affected their smoking behaviour. Psychological and behavioural interventions can promote smoking control management and encourage drivers to quit or limit smoking. Online car-hailing companies can also establish a complaint mechanism combined with personal credit.

**Supplementary Information:**

The online version contains supplementary material available at 10.1186/s12889-021-11366-8.

## Introduction

Online ride-hailing refers to the business activities of providing non-cruise booking taxi service based on a service platform that integrates information of supply and demand relying on Internet technology [1]. Compared with the traditional taxi, it has the advantages of high efficiency, good experience, convenience, and low price [2, 3]. Online ride-hailing services are rapidly growing in many countries. Currently, DiDi, Grab, Lyft, Ola, and other ride-hailing platforms have covered thousands of cities and built a global network [4]. In China, Didi Chuxing Technology Co., as the largest mobile travel platform, provided over 7.43 billion mobile trips to 450 million passengers in more than 400 cities in 2017 [5]. As an emerging travel mode, online ride-hailing plays an increasingly significant role in people’s daily travel, with a user volume of 362 million in China by March 2020 [6].

A wide-reaching survey in China reported that the occupational group of taxi drivers generally has a high smoking rate [7–9] due to various factors, such as long working hours, high intensity of work and the need to refresh themselves when they are tired [10, 11]. Regular smoking can cause various malignant tumours and respiratory system, cardiovascular and cerebrovascular system and other systemic diseases. In addition, smoking in a narrow and closed carriage imposes health risks to passengers and danger during driving [12].

In 2011, the detailed rules for the *Regulations on the Administration of Sanitation at Public Places* issued by the Ministry of Health of the People’s Republic of China stipulated for the first time the contents of smoking control in public transport. In accordance with our review of local government documents, at present, 90% of Chinese provincial administrative regions have issued policies and regulations banning smoking in public transport. However, the definition of public transport varies from city to city, and only a few provinces, such as Beijing and Sichuan Province, have listed taxis as non-smoking public vehicles. The nature of online car-hailing is between a traditional taxi and private car [1]. Its position is not clear, and the existing smoking ban policy fails to restrain it, making it a blind spot for anti-smoking management. Prior research showed that high smoking rates could lead to low compliance with tobacco regulations [13]. Coupled with incomplete national policies on smoking control in online ride-hailing vehicles, second-hand smoke exposure in the cars is inevitable. Thus, the smoking status and related factors of the occupational group of ride-hailing drivers require further investigations. Yet, relevant studies remain lacking.

Local and international studies have determined various influencing factors of adult smoking, including gender [14–17], marital status [18, 19], educational level [20], economic level [21], social networks [22, 23], health status [24, 25], mental illness [26–28], alcohol/drug abuse and dependence [29–32], race/ethnicity [33] and work pressure [34, 35], etc. Moreover, Hu et al. [36] and Li et al. [37] pointed out that the effect of skipping breakfast, short sleep duration, and participation in recreational activities and physical exercises on the smoking behaviour of rural residents was significant. Specific factors influence a certain population. For example, prior studies showed that job-related stressors affected the increase of smoking intensity and nicotine dependence of American military personnel [38]; the smoking behaviour of spouses affected the smoking during pregnancy of women [39]; shift work affected the smoking of manufacturing workers [40]. At present, only a few investigated the factors influencing drivers’ smoking. Norman GJ et al. [41] and Josh Martin et al. [42] reported that households and regions with low economic level had associations with low smoke exposure rate in cars. By contrast, households and regions with high economic levels were related to high smoke exposure rates in cars. Jain NB et al. [43] showed that job title, education level, residential area, scale and location of truck transport terminals were determinants of smoking amongst truck transport drivers. Ozoh OB et al. [44] studied the prevalence and related factors of smoking amongst commercial long-distance drivers in Lagos, Nigeria. They found that smoking friends, freight driving and low educational level were the factors affecting current smoking. To sum up, the factors influencing smoking in drivers, such as demographic and socioeconomic characteristics, are highly similar to those in adults. The occupational characteristics of drivers themselves can also affect their smoking behaviour.

On the basis of the above discussion, we designed a survey questionnaire for online ride-hailing drivers in China. The current developments of online ride-hailing provide a convenient way to conduct large scale online survey. Therefore, using a platform of ride-hailing drivers, we surveyed a large sample, covering the vast majority of areas with available ride-hailing services (31 provincial-level administrative regions). The analysis results offer a comprehensive perspective on the smoking problems of ride-hailing drivers. They contribute to the understanding of different socio-economic and working characteristics that affect the smoking status of drivers. The risk factors for smoking behaviour based on the large random sample study can provide real-world evidence for international tobacco control researchers.

## Methods

### Study design and participants

From September to November 2017, using the platform of ride-hailing companies with 91% market share in China, we distributed questionnaires to drivers’ accounts through the Wenjuanxing platform (https://www.wjx.cn/), which provides functions equivalent to Amazon Mechanical Turk. A total of 9003 questionnaires were assigned, of which 8990 were considered valid, with an effective rate of 99.86%. For large populations of more than 150,000, a sampling ratio of 1%, or approximately 1500 sample sizes, will obtain the correct result [45]. Considering that the full-time ride-hailing drivers investigated in this study belong to a large population, our sample size could achieve ideal accuracy.

### Measures

The design of the questionnaire referred to the previous literature on the influence factors of smoking. We also considered the questionnaires of the National Health Service Survey and the National Population Census. The final version passed the demonstration of experts in health management and disease prevention and control. The questionnaire had four parts: sociodemographic characteristics, job characteristics, health-related factors and smoking status. The health-related factors included healthy behaviour, health status and health literacy of three aspects (Table 1).

**Table 1:** Questions and variable settings in the survey questionnaire

Dimension	Variables	Assignment
**Smoking status**	Have you smoked in the past 30 days?	Yes = 1; No = 0
	Have you smoked in the car?	Yes = 1; No = 0
	Do you permit passengers to smoke in the car?	Yes = 1; No = 0
**Sociodemographic characteristics**	Area of residence*	Eastern regions = 1; Central regions = 2; Western regions = 3
	Gender	Male = 1; Female = 2
	Age	18–34 years = 1; 35–44 years = 2; ≥45 years = 3
	Marital status	Married = 1; Unmarried = 2; Divorced/Widowed/Remarried/Others = 3
	Educational level	Junior high school and below = 1; Vocational/Technical secondary school/High school = 2; Junior college and above = 3
	The ratio of family burden†	Less than the gross dependency coefficient‡ = 1; Equal or more than the gross dependency coefficient = 2
**Job characteristics**	Working years as a full-time driver	< 5 years = 1; 5–10 years = 2; ≥10 years = 3
	Working hours per day	< 10 h = 1; 10–12 h = 2; ≥12 h = 3
	Period of work	Daytime = 1; Night = 2; Both daytime and night = 3; Non-fixed working time = 4
	Time spent per day outside the car to relax during work	< 5 min = 1; 5–10 min = 2; ≥10 min = 3
**Health-related factors**	**Health status**	
	Experiences of chronic illness§	Yes = 1; No = 0
	Self-reported health	Good = 1; General = 2; Poor = 3
	Feelings of anxiety	Never = 1; Occasionally = 2; Frequently = 3
	Weight status¶	Underweight = 1; Normal = 2; Overweight = 3
	**Health behaviour**	
	Drinking status	Current drinker = 1; Nondrinker = 0
	Eating habits	Extremely regular = 1; Regular in general = 2; Extremely irregular = 3
	Choice of place to eat	Mainly eating at home = 1; Mainly eating out = 2
	Sleep duration per day	< 6 h = 1; 6–8 h = 2; ≥8 h = 3
	The number of days in a week of engaging in at least 10 min of moderate or vigorous exercise	0 day = 1; 1–2 days = 2; ≥3 days = 3
	**Health literacy**	
	Level of the initiative to acquire health knowledge	Never = 1; Occasionally = 2; Frequently = 3
	Main approaches for acquiring health knowledge	Mobile phone; Television shows; Broadcast; Internet; Doctors; Newspapers; Families/Colleagues/Friends; Brochure or bulletin board; Others

*Following the classification standards commonly used in national statistics, the eastern, central and regional areas include 11, 8 and 12 provincial administrative regions, respectively [46]

†The original questions were ‘the number of families’ and ‘the number of children and the elderly in the family’. We converted them into the indicator ‘the ratio of family burden’ following the formula ‘the ratio of family burden = (the number of people aged 14 and under + the number of people aged 65 and over)/ the number of people aged 15–64 × 100%’

‡On the basis of the number of Chinese people of all ages at the end of 2017, the gross dependency coefficient was 41.19%

§The chronic illness was not self-reported but medically diagnosed

¶Weight status was classified using body mass index (BMI): BMI between 18.5 and 23.9 kg/m^2^ was considered normal weight, less than 18.5 kg/m^2^ was underweight and higher than 23.9 kg/m^2^ was overweight

### Quality control

In the process of the questionnaire design, we tested the logic of the questions. We used Cronbach’s PCR coefficient and semi-correlation to test the reliability of the scale. The results showed that the total Cronbach’s *p* coefficient of the scale was 0.899, and the semi-correlation coefficient was 0.833. Except for age, all questions adopted a closed questionnaire design. The questionnaire could not be submitted until all the questions were finished, and the same account could only fill in the questionnaire once. The data was directly exported from the electronic questionnaire database, avoiding manual input errors. To rule out errors in the filling, we cleaned the data to eliminate invalid questionnaires. Specifically, we excluded those with contradicting answers to different questions. For example, the chronic disease reported by the respondents did not match their age or gender, and the self-reported age did not qualify for the given years in service.

### Statistical analysis

Numerical data were converted into categorical variables and displayed with numbers and percentages. We analysed the correlation between current smokers and other indicators by conducting a chi-square test. We used binary logistic regression to investigate the mixed effect of the smoking behaviour of drivers. We used the respondents’ smoking status as the dependent variable and the statistically significant correlation factors found in the chi-square test as the independent variables. We also included multiple categorical variables in the model as dummy variables. We considered a two-tailed *P*-value < 0.05 and ORs with 95% CIs for the significant relationship between the exposure and outcome variables. We conducted all analyses using SPSS software version 22.0.

## Results

### Characteristics of the drivers

Table 2 shows the characteristics of the respondents. A total of 5133 cases (57.1%) were from the eastern regions, 1812 cases (20.2%) were from the central regions, and 2045 cases (22.7%) were from the western regions with the highest smoking rate (60.1%, *P* < 0.001). The proportion of male and female drivers was 95.9 and 4.1%, respectively, and the smoking rate of male drivers was significantly higher than that of female drivers (56.7% *v* 37.6%, *P* < 0.001). Moreover, 43.8, 31.4 and 24.8% of the drivers were working for < 10 h a day, 10–12 h a day and > 12 h a day. The longer the working hours, the higher the smoking rate (*P* = 0.005). Those who reported poor physical (*P* = 0.012) and mental health (*P* < 0.001) had a higher smoking rate than other drivers. Smoking habits were common amongst drivers who had unhealthy daily habits and behaviours, such as drinking alcohol (*P* < 0.001), irregular diet (*P* < 0.001), mainly eating out (*P* < 0.001), short sleep duration (*P* < 0.001) and little exercise time (*P* < 0.001).

**Table 2:** Smoking status by different characteristics

Variables	Total n (%)	Current non-smoker n (%)	Current smoker n (%)	*P-value*
**Area of residence**				< 0.001
Eastern regions	5133 (57.1)	2377 (46.3)	2756 (53.7)	
Central regions	1812 (20.2)	774 (42.7)	1038 (57.3)	
Western regions	2045 (22.7)	815 (39.9)	1230 (60.1)	
**Gender**				< 0.001
Male	8623 (95.9)	3737 (43.3)	4886 (56.7)	
Female	367 (4.1)	229 (62.4)	138 (37.6)	
**Age**				< 0.001
18–34 years	3054 (34.0)	1248 (40.9)	1806 (59.1)	
35–44 years	3941 (43.8)	1782 (45.2)	2159 (54.8)	
≥ 45 years old	1995 (22.2)	936 (46.9)	1059 (53.1)	
**Marital status**				0.107
Unmarried	610 (6.8)	266 (43.6)	344 (56.4)	
Married	7662 (85.2)	3428 (44.7)	4234 (55.3)	
Divorced/Widowed/Remarried/Others	718 (8.0)	272 (37.9)	446 (62.1)	
**Educational level**				0.827
Junior high school and below	2495 (27.8)	1122 (45.0)	1373 (55.0)	
Vocational/Technical secondary school/High school	4635 (51.6)	1995 (43.0)	2640 (57.0)	
Junior college and above	1860 (20.7)	849 (45.6)	1011 (54.4)	
**The ratio of family burden**				0.244
Less than the gross dependency coefficient	1220 (13.6)	1906 (44.5)	2374 (55.5)	
Equal or more than the gross dependency coefficient	7770 (86.4)	2060 (43.7)	2650 (56.3)	
**working years as a full-time driver**				0.399
< 5 years	6019 (67.0)	2662 (44.2)	3357 (55.8)	
5–10 years	1501 (16.7)	679 (45.2)	822 (54.8)	
> 10 years	1470 (16.4)	625 (42.5)	845 (57.5)	
**working hours per day**				0.005
< 10 h	3939 (43.8)	1801 (45.7)	2138 (54.3)	
10–12 h	2822 (31.4)	1226 (43.4)	1596 (56.6)	
> 12 h	2229 (24.8)	939 (42.1)	1290 (57.9)	
**Period of work**				< 0.001
Daytime	2581 (28.7)	1282 (49.7)	1299 (50.3)	
Night	246 (2.7)	84 (34.1)	162 (65.9)	
Both daytime and night	4624 (51.4)	1959 (42.4)	2665 (57.6)	
Non-fixed working time	1539 (17.1)	641 (41.7)	898 (58.3)	
**Time spent per day outside the car to relax during work**				0.266
< 5 min	4355 (48.4)	1952 (44.8)	2403 (55.2)	
5–10 min	3270 (36.4)	1419 (43.4)	1851 (56.6)	
> 10 min	1365 (15.2)	595 (43.6)	770 (56.4)	
**Experiences of chronic illness**				0.993
Yes	6240 (69.4)	2753 (44.1)	3487 (55.9)	
No	2750 (30.6)	1213 (44.1)	1537 (55.9)	
**Self–reported health**				0.012
Good	2831 (31.5)	1308 (46.2)	1523 (53.8)	
General	3637 (40.5)	1577 (43.4)	2060 (56.6)	
Poor	2522 (28.1)	1081 (42.9)	1441 (57.1)	
**Feelings of anxiety**				< 0.001
Never	3225 (35.9)	1511 (46.9)	1714 (53.1)	
Occasionally	3546 (39.4)	1534 (43.3)	2012 (56.7)	
Frequently	2219 (24.7)	921 (41.5)	1298 (58.5)	
**Current drinker**				< 0.001
Yes	4325 (48.1)	1610 (37.2)	2715 (62.8)	
No	4665 (51.9)	2356 (50.5)	2309 (49.5)	
**Eating habits**				< 0.001
Extremely regular	2621 (29.2)	1332 (50.8)	1289 (49.2)	
Regular in general	1924 (21.4)	922 (47.9)	1002 (52.1)	
Extremely irregularly	4445 (49.4)	1712 (38.5)	2733 (61.5)	
**Choice of place to eat**				< 0.001
mainly eating at home	3795 (42.2)	1808 (47.6)	1987 (52.4)	
mainly eating out	5195 (57.8)	2158 (41.5)	3037 (58.5)	
**Sleep duration per day**				< 0.001
< 6 h	2407 (26.8)	957 (39.8)	1450 (60.2)	
6–8 h	5737 (63.8)	2608 (45.5)	3129 (54.5)	
> 8 h	846 (9.4)	401 (47.4)	445 (52.6)	
**The number of days in a week of engaging in at least 10 min of moderate or vigorous exercise**				< 0.001
0 day	5281 (58.7)	2185 (41.4)	3096 (58.6)	
1–2 days	2803 (31.2)	1285 (45.8)	1518 (54.2)	
≥ 3 days	906 (10.1)	496 (54.7)	410 (45.3)	
**Level of the initiative to acquire health knowledge**				< 0.001
Never	1724 (19.2)	647 (37.5)	1077 (62.5)	
Occasionally	4471 (49.7)	1864 (41.7)	2607 (58.3)	
Frequently	2795 (31.1)	1455 (52.1)	1340 (47.9)	
**Weight status**				< 0.001
Underweight	394 (4.4)	139 (35.3)	255 (64.7)	
Normal weight	3815 (42.4)	1619 (42.4)	2196 (57.6)	
Overweight	4781 (53.2)	2208 (46.2)	2573 (53.8)	

The rate of current smoking amongst the respondents was 55.9%. Nearly one-third of smokers smoked in their cars (32.2%, Table 3).

**Table 3:** Smoking problems amongst drivers

		Total n (%)	Smoking in the car, n (%)	
			Yes	No
Current smoker	Yes	5024 (55.9)	1618 (32.2)	3406 (67.8)
	No	3966 (44.1)	–	–

### Multivariate logistic regression analysis results

The logistic regression results were as follows (Table 4): gender, area of residence, the period of work, drinking status (whether a current drinker), eating habits, the number of days a week of engaging in at least 10 min of moderate or vigorous exercise, level of the initiative to acquire health knowledge and weight status were the relevant influencing factors of current smoking (*P* ≤ 0.001). Amongst them, current drinker (OR = 1.663, 95% CI [1.526, 1.813]), extremely regular eating habits (OR = 1.370, 95% CI [1.233, 1.523]) and underweight (OR = 1.249, 95% CI [1.001, 1.559]) were the risk factors of current smoking. The drivers in the central (OR = 1.172, 95% CI [1.049, 1.309]) and western regions (OR = 1.330, 95% CI [1.194, 1.480]) had a higher risk of smoking than those in eastern regions, and the smoking risk of the drivers who drove at night (OR = 1.938, 95% CI [1.463, 2.568]) or both daytime and night (OR = 1.287, 95% CI [1.164, 1.424]) or non-fixed time (OR = 1.393, 95% CI [1.223, 1.588]) was greater than that of day-shift drivers. Female (OR = 0.519, 95% CI [0.416, 0.647]), exercising at least three days per week (OR = 0.752, 95% CI [0.646, 0.875]), occasionally (OR = 0.882, 95% CI [0.783, 0.992]) or frequently acquiring health knowledge (OR = 1.393, 95% CI [1.223, 1.588]) and overweight (OR = 0.675, 95% CI [0.591, 0.770]) were protective factors for current smoking.

**Table 4:** Factors influencing smoking behaviour of online ride-hailing drivers

Variables	OR* (95% CI**)	*P-value*
**Gender**		< 0.001
Male	Reference	
Female	0.519 (0.416 to 0.647)	
**Area of residence**		< 0.001
Eastern regions	Reference	
Central regions	1.172 (1.049 to 1.309)	0.005
Western regions	1.330 (1.194 to 1.480)	< 0.001
**Period of work**		< 0.001
Daytime	Reference	
Night	1.938 (1.463 to 2.568)	< 0.001
Both daytime and night	1.287 (1.164 to 1.424)	< 0.001
Non-fixed working time	1.393 (1.223 to 1.588)	< 0.001
**Drinking status**		< 0.001
Nondrinker	Reference	
Current drinker	1.663 (1.526 to 1.813)	< 0.001
**Eating habits**		< 0.001
Extremely regular	Reference	
Regular in general	1.071 (0.949 to 1.208)	0.269
Extremely irregular	1.370 (1.233 to 1.523)	< 0.001
**The number of days in a week of engaging in at least 10 min of moderate or vigorous exercise**		0.001
0 day	Reference	
1–2 days	0.987 (0.893 to 1.091)	0.800
≥ 3 days	0.752 (0.646 to 0.875)	< 0.001
**Level of the initiative to acquire health knowledge**		< 0.001
Never	Reference	
Occasionally	0.882 (0.783 to 0.992)	0.037
Frequently	0.675 (0.591 to 0.770)	< 0.001
**Weight status**		< 0.001
Normal weight	Reference	
Underweight	1.249 (1.001 to 1.559)	0.049
Overweight	0.846 (0.775 to 0.924)	< 0.001

Note: (1) The modelling test of the logistic regression:

Hosmer and Lameshow’s *P*-value = 0.767 (*P* > 0.05)

−2LL = 11,875.540 (*P* < 0.001)

Cox and Snell’s *R*^2^ = 0.050

(2) *OR, odds ratio; **CI, confidence interval

## Discussion

### Smoking behaviour of drivers

The prevalence of smoking amongst ride-hailing drivers in our survey was much higher than that of Chinese adults (26.6% [47]). It was consistent with that of taxi drivers in other large surveys across the country [7–9]. Many smokers also smoked in cars, forcing passengers to breathe second-hand smoke. This situation poses health risks and not conducive to the construction of urban civilisation.

### Sociodemographic characteristics and smoking

Gender had a major influence on the current smoking of online ride-hailing drivers, which was consistent with the smoking pattern in most studies [48–50]. Some men see smoking as a sign of masculinity [51], and Chinese society, still influenced by a partly rooted tradition, is tolerant of male smoking but not female smoking [52]. Thus, smoking is more prevalent in men than in women.

Chinese drivers’ smoking behaviour also reflected regional differences. Smoking poses higher risks amongst the drivers in the central and western regions than those in the eastern regions. The *Report on the Current Situation of Smoking in China in 2016* [53] showed that the central and western regions and Yunnan Province had the highest proportion of smokers, followed by the eastern coastal areas. The distribution of smoking amongst online taxi-hailing drivers was similar. Given the various reasons, the influence of smoking culture has the most effect. Smoking culture in China has a long history, and it varies from place to place due to vast Chinese territory. Northeast tobacco and Hunan tobacco are typical examples, where the proportion of smokers in China is also at a high level. The central and western regions are also known for their prominent tobacco industries. As a big part of their regional economies, such industries will have an effect to some extent. Finally, the strictness of policies and regulations in different regional areas is varied. The eastern regions are more developed, with a relatively complete smoke-free policy and stricter implementation. Therefore, the proportion of smokers in the eastern regions has been declining compared with the central and western regions in recent years [54].

Unlike day-shift drivers, working at night, working at both daytime and night and working at the non-fixed time were risk factors for drivers to smoke. Some ride-hailing platforms need to ensure a certain amount of time online every day, and due to the uncertainty of the orders, the driver needs to keep an eye on the phone. Full-time ride-hailing drivers tend to work long hours with relatively little freedom. More than half (56.2%, Table 2) of the respondents worked more than 10 h a day, and 71.3% usually drove at night, both daytime and night or an irregular time. Studies have shown that shift work can cause the disorder of human biological rhythm, which harms health. Bad behaviours, such as smoking and drinking, are common amongst shift workers [55], and drivers may use smoking to refresh themselves and relieve fatigue.

### Health behaviour and smoking

Our analysis results showed that more exercise could reduce smoking risk, which was consistent with prior findings on the association between physical activity and smoking [56]. Studies have shown that exercise can reduce smoking desire and withdrawal response to smoking cessation and ultimately help people stop smoking [57–59]. The mechanism may be that exercise and smoking have similar effects on neurobiological processes, such as increasing the levels of beta-endorphin in smokers, to serve as an alternative reinforcer of smoking [60, 61].

Drivers with extremely irregular eating habits had a higher risk of smoking than those with extremely regular eating habits, which was consistent with the results of Doris Anzengruber et al. [62] on the relationship between smoking and irregular diet. Smoking behaviour was common in individuals with irregular diets and associated with impulsive personality traits in groups with eating disorders, such as overeating.

In the current study, we found that current drinkers had a higher risk of smoking than nondrinkers. Johannes Thrul et al. [63] pointed out that alcohol and tobacco were often used together, and smoking whilst drinking could increase pleasure. Thus, drinking might inhibit successful smoking cessation. For drivers, alcohol will directly damage their health and increase the risk of other health hazards and be one of the main culprits of traffic accidents. In 2011, drunk-driving was included in criminal law in China. Thus, strengthening drivers’ perception of the risks brought by drinking is crucial. Understanding this matter will help them control their drinking behaviour.

### Health literacy and smoking

The health awareness of the ride-hailing drivers was weak, which needs to be improved. Nearly half of the drivers sought health knowledge only when they were sick or not feeling well (49.7%, Table 2). Less than one-third often actively acquired health knowledge. Health literacy can promote healthy behaviour in the general population and reduce the risk of addiction to unhealthy behaviours. People with high health literacy will be more aware of the hazards associated with smoking and pay more attention to their health, leading to a low smoking rate. This statement is consistent with the theoretical model of Knowledge, Trust and Practice (KPA) in health education.

### Obesity and smoking

More than half of the ride-hailing drivers (53.2%, Table 2) were overweight, much higher than the national average of 42.1% [64]. Some studies have shown that obesity and smoking, both as influencing factors of various diseases, can increase the risk of cardiovascular and cerebrovascular diseases amongst drivers [65, 66]. The relationship between smoking and obesity is extremely complicated. Current studies generally agree that smoking can reduce obesity because smoking suppresses appetite at the cellular level and reduces energy intake and consumption [67]. Other studies suggest that obese people may lose weight by starting to smoke. Consequently, it may increase nicotine dependence and influence smoking intensity [68]. However, through Mendel randomised studies with large samples, Carreras-Torres et al. [69] found that the increase in BMI had a high correlation with current smoking status and smoking intensity. Such a high association may be caused by clusters of single nucleotide polymorphisms in neuronal pathways, suggesting that addictive behaviours, such as nicotine addiction, and high energy intake may have a common biological basis. Thus, the causal relationship between obesity and smoking requires further investigations.

Weight may also temporarily increase in the process of quitting smoking. Thus, some smokers, especially women, do not try to or fail to give up smoking [70].

### Limitations

This study has several limitations. Firstly, numerous complex factors affect smoking behaviour. We could only discuss the main factors by combining our literature review with the characteristics of the respondents. Thus, neglecting some possible influencing factors is difficult to avoid. Secondly, given the limitations of cross-sectional studies, we could not determine causal relationships between certain variables and smoking outcomes. We only inferred such relationships from similar studies. Thirdly, we adopted an electronic self-filling questionnaire. Such a method could not ensure the authenticity and independent completion of the respondents. Accordingly, we guaranteed the quality of the questionnaire through a standardised questionnaire option design and online car-hailing platform system monitoring.

## Conclusion

Our survey showed that the smoking rate of online ride-hailing drivers was high, demanding immediate action. Policymakers and company managers can implement tobacco control amongst online ride-hailing drivers from two aspects. Firstly, online ride-hailing drivers should be encouraged to stop or limit smoking. On the one hand, they should undergo psychological intervention, especially the male drivers in the central and western regions. Company managers should consider adding ‘No Smoking’ signs in the car and setting up health promotion columns on the driver and passenger interface of online ride-hailing apps. Such materials can remind and inform the drivers about the health risks of smoking. In this way, drivers can stop relying on misleading information available online. On the other hand, behavioural intervention should guide drivers to exercise more, eat a balanced meal and maintain a healthy lifestyle. Alcohol restriction intervention and weight intervention should also assist in tobacco cessation or restriction. Secondly, authorities in the ride-hailing industry can set up convenient channels for complaints as part of the credit rating system amongst drivers and riders.

## Supplementary Information

**Additional file 1.** A questionnaire on the health status and behavior of online ride-hailing drivers in China.

## Data Availability

The datasets used and analysed during the current study are available from the corresponding author on reasonable request.
