# Supplementary material for: Factors influencing smoking behaviour of online ride-hailing drivers in China: a cross-sectional analysis
Source: BMC Public Health. 2021 Jul 6;21:1326. doi: 10.1186/s12889-021-11366-8 (PMC8259384; doi:10.1186/s12889-021-11366-8)
Supplement: Supplementary file 1 — Additional file 1. A questionnaire on the health status and behavior of online ride-hailing drivers in China. [file 12889_2021_11366_MOESM1_ESM.docx]

**Title page**

Title of paper:

**The influencing factors of the smoking behavior of online ride-hailing drivers in China: A cross-sectional analysis**

Authors

| Name | Degree | Affiliation | Email address |
| --- | --- | --- | --- |
| Xinlin Chen | BA | School of Public Health, Capital Medical University, Fengtai District, Beijing, China | chenxinlin@hust.edu.cn |
| Xuefei Gu | PhD | China National Health Development Research Center | gu.xuefei@foxmail.com |
| Tingting Li | MA | China National Health Development Research Center | litt@nhei.cn |
| Lirong Xu | BA | School of Public Health, Capital Medical University, Fengtai District, Beijing, China | xulirong150607@sina.com |
| Qiaoyan Liu | PhD | China Rehabilitation Research Center, Beijing, China | liuqiaoyancr@gmail.com |
| Bo Peng | MA | Institute of Medical Information，China Academy of Chinese Medical Sciences，Beijing | peng_boo@126.com |
| Nina Wu* | PhD | School of Public Health, Capital Medical University, Fengtai District, Beijing, China | wunina@ccmu.edu.cn |

**Corresponding author**

Dr. Nina Wu

Current address: No 10 Xitoutiao, Youanmenwai Street, Fengtai District, Beijing, China

Telephone number: +86 10 83911578

Fax number: +86 10 83911602

Email address: wunina@ccmu.edu.cn

**A questionnaire on the health status and Behavior of online ride-hailing drivers in China**

| **1.Sociodemographic characteristics** | |  |
| --- | --- | --- |
|  | Family size: (1) 1 (2) 2 (3) 3 (4) 4 (5) 5 (6) ≥6 |  |
|  | Number of children under 16 years of age and over 65 years of age living together: (1) 1 (2) 2 (3) 3 (4) 4 (5) 5 (6) ≥6 |  |
|  | Gender: (1) Male (2) Female |  |
|  | Age: (1)15-24 (2)25-34 (3) 35-44 (4) 45-54 (5) 55-64 (6) 65- |  |
|  | Marital status:(1) Unmarried (2) Married (3) Remarried (4) Divorced (5) Widowed (6) Others |  |
|  | Education: (1) Junior high school and below (2) high school/ technical school (3) technical secondary school/ secondary technical school (4) junior college (5) Undergraduate (6) master’s degree or above |  |
|  | What kind of social health insurance do you currently participate in?  (1) basic medical insurance for urban employees (2) basic medical insurance for urban residents (3) New rural cooperative medical care (4) other social medical insurance (5) Did not participate |  |
| **2. Work characteristics** | |  |
|  | How many years have you been a full-time driver?  (1) < 3 years (2)3-5 years (3)5-10 years (4)10-20 years (5) ≥ 20 years |  |
|  | How many hours do you usually work every day?  (1) <8 hours (2)8-10 hours (3)10-12 hours (4)12-14 hours (5) > 14 hours |  |
|  | Period of work:  (1) daytime (2) night (3) both daytime and night (4) non-fixed working time |  |
|  | How many days a week do you usually take off?  (1) none (2) 1 day (3)2 days (4)3 days (5) ≥ 4 days |  |
|  | How long do you spend outside the car for relax during work per day (e.g., stretching your body and turning your head and neck)?  (1) none (2) 5-10 minutes per day (3) 10-30 minutes per day (4) ≥ 30 minutes per day |  |
|  | How much water do you drink each day?  (1) < 200 ml (2)200-400 ml (3)400-800 ml (4) ≥ 800 ml |  |
| **3. Disease and injury** | |  |
|  | Whether you have **the following chronic diseases** that have been diagnosed by doctors? (multiple choice)  (1) hypertension (2) hyperlipidemia (3) diabetes (4) cardiovascular disease (5) fatty liver (6) tumors (7) chronic respiratory diseases (8) cervical / lumbar vertebral disease (9) arthritis (10) prostatitis/ gynecological inflammation (11) hemorrhoids (12) gastrointestinal diseases (13) kidney disease (14) chronic conjunctivitis  (15) Other chronic diseases (16) none (17) cystitis |  |
|  | How many times have you been to hospital due to illness in the past 12 months? (1) none (2) once (3) 2 times (4) ≥ 3 times |  |
| **4. Health status and behavior** | |  |
|  | Self-reported health:  (1) very good (2) good (3) generally (4) not very good (5) very bad |  |
|  | How often have you felt anxiety recently?  (1) I don't feel (2) two or three times a year (3) two or three times a month  (4) two or three times a week (5) every day |  |
|  | Are you under any of the following conditions while driving? (multiple options)  (1) fidgeting in traffic jams (2) more irritable than usual while driving  (3) block someone from entering your lane while driving (4) swearing more often than usual while driving (5) none |  |
|  | Have you had a health check-up in the last 12 months? (Excluding tests made due to illness) (1) yes (2) No |  |
|  | When did you last have your blood pressure measured?  (1) within 1 month (2) 2-3 months (3) 4-6 months (4) 6-12 months  (5) 12 months ago |  |
|  | Do you eat regular meals on time?  (1) Very regular (2) regular most of the time  (3) Occasionally irregular (4) very irregular |  |
|  | How often do you eat out?  (1) eat at home three times a day (2) most meals are eaten at home  (3) most meals are eaten out (4) eat out three times a day |  |
|  | The most important factor to consider when you eat out: (single choice)  (1) affordability (2) adequate weight (3) nutritional balance (4) saving time (5) hygiene (6) parking convenience (7) others |  |
|  | How long do you sleep every day?  (1) < 4 hours (2) 4-6 hours (3) 6-8 hours (4)8-10 hours (5) ≥ 10hours |  |
|  | Have you smoked in the last 30 days? (1) Yes (2) No |  |
|  | Do you smoke in the car? (1) Yes (2) No |  |
|  | Do you allow passengers to smoke in the car? (1) Yes (2) No |  |
|  | Have you been drinking alcohol for the past 30 days? (1) Yes (2) No |  |
|  | How often have you been drinking in the last 12 months? (1) More than 6 days per week (2) 5-6 days per week (3) 3-4 days a week (4) 1-2 days a week (5) 1-3 days per month (6) less than 1 day per month |  |
|  | How many days do you engage in at least 10 minutes of moderate or vigorous exercise? (e.g., medium and high intensity housework activities, recreational physical activities)  (1) none (2) 1-2 days (3) 3-4 days per week (4) more than 5 days per week |  |
|  | In addition to driving, how long do you spend sitting quietly watching TV, using a computer, playing video games, reading, etc. in your spare time every day?  (1) 1 hour (2) 2 hours (3) 3 hours (4) 4 hours (5)5 hours (6) 6 hours or more |  |
|  | Do you take the initiative to acquire health knowledge?  (1) Never (2) occasionally (when sick and uncomfortable) (3) often |  |
|  | Where do you usually get health information? (multiple options)  (1) mobile phone (2) TV (3) broadcast (4) Internet (5) newspapers and books  (6) families/ colleagues/ friends (7) doctors (8) pictures/ bulletin board (9) Others |  |
|  | What health-related information would you like? (multiple options)  (1) Risk of disease and prevention (2) symptoms of the disease and diagnosis  (3) treatment of the disease and effectiveness (4) nutritional diet and home nursing (5) fitness and exercise guidance (6) health care knowledge (7) hospital and specialty introduction (8) mental health knowledge or counseling (9) others |  |
|  | What is your height? (cm) |  |
|  | What's your weight? (kg) |  |
|  | What's your city? (There are administrative divisions and province codes.) |  |
